# Rotational manipulation of single cells and organisms using acoustic waves

**DOI:** 10.1038/ncomms11085

**Published:** 2016-03-23

**Authors:** Daniel Ahmed, Adem Ozcelik, Nagagireesh Bojanala, Nitesh Nama, Awani Upadhyay, Yuchao Chen, Wendy Hanna-Rose, Tony Jun Huang

**Affiliations:** 1 Department of Engineering Science and Mechanics, The Pennsylvania State University, University Park, Pennsylvania 16802, USA; 2 Department of Biochemistry and Molecular Biology, The Pennsylvania State University, University Park, Pennsylvania 16802, USA; 3 Department of Bioengineering, The Pennsylvania State University, University Park, Pennsylvania 16802, USA

## Abstract

The precise rotational manipulation of single cells or organisms is invaluable to many applications in biology, chemistry, physics and medicine. In this article, we describe an acoustic-based, on-chip manipulation method that can rotate single microparticles, cells and organisms. To achieve this, we trapped microbubbles within predefined sidewall microcavities inside a microchannel. In an acoustic field, trapped microbubbles were driven into oscillatory motion generating steady microvortices which were utilized to precisely rotate colloids, cells and entire organisms (that is, *C. elegans*). We have tested the capabilities of our method by analysing reproductive system pathologies and nervous system morphology in *C. elegans*. Using our device, we revealed the underlying abnormal cell fusion causing defective vulval morphology in mutant worms. Our acoustofluidic rotational manipulation (ARM) technique is an easy-to-use, compact, and biocompatible method, permitting rotation regardless of optical, magnetic or electrical properties of the sample under investigation.

## Abstract (web summary)

The precise rotational manipulation of single cells is technically challenging and relies on the optical, magnetic and electrical properties of the biospecimen. Here the authors develop an acoustic-based, on-chip manipulation method that can rotate single microparticles, cells and organisms.

## Graphical abstract

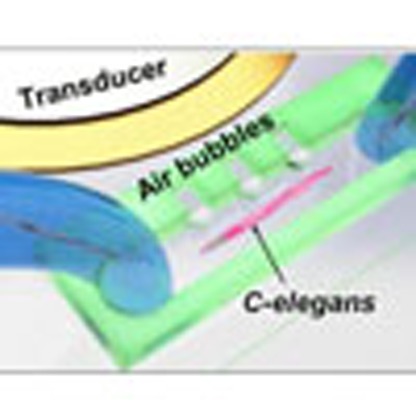

Precise rotational manipulation of particles, cells and multicellular organisms is an essential capacity in biotechnology that impacts various disciplines including single-cell analysis^1^,^2^, drug discovery^3^,^4^ and organism studies^5^,^6^,^7^. For example, distinct rotational behaviour due to different cell morphologies has been identified as a potential diagnosis method^8^,^9^. Providing three-dimensional (3D) interrogation, rotational manipulation can reveal hidden genetic, cellular and structural details that are vital in small organism phenotyping^10^,^11^,^12^,^13^, screening^6^,^14^,^15^ and microsurgery^16^,^17^,^18^ and which are not visible in typical translational manipulation.

Although several techniques have been established for the translational manipulation of particles and cells (for example, optical tweezers^19^, magnetic tweezers^20^, acoustic tweezers^21^,^22^, dielectrophoresis^23^ and electrorotation^24^,^25^), performing rotational manipulation has remained a significant challenge. Optical tweezers are widely used for generating rotational manipulation but the results are limited by physiological damage to cells and other biospecimens due to laser-induced heating^26^. In addition, the manipulation capability of existing methods strongly depends on the optical^27^,^28^, magnetic^9^,^29^ and electrical^30^,^31^ properties of the biospecimen. Recent studies have demonstrated acoustic vortex generation by an array of individually addressable transducers excited at various phases^32^,^33^,^34^, by acoustic transducers with helicoidal wave radiating surfaces^35^, and by the optoacoustic technique^36^. However, as of yet, no existing method has demonstrated the capacity to rotate small model animals (such as *Caenorhabditis elegans*) or cells on-chip.

Acoustofluidic methods that fuse acoustics and microfluidics have the potential for dramatically improving methods for manipulating cells and small animals. Acoustically driven trapped microbubbles are important tools in microfluidics^37^ that have been used in various applications including microflow control^38^, fluid mixing^39^ and pumping^40^, particle manipulation^41^,^42^,^43^ and gradient and chemical waveform generation^44^,^45^. In this article, we describe an acoustofluidic rotational manipulation (ARM) method, which utilizes steady acoustic microstreaming vortices created by the oscillatory motion of air microbubbles trapped in a microfluidic channel. The oscillatory motion is driven by the application of a low-power acoustic field. The ARM method presented here represents the first acoustic-based rotational manipulation approach to rotate biospecimen. This method is extremely versatile. Its operation is independent of the samples' intrinsic properties. It allows effective and precise rotation of specimens over a wide range of sizes, shapes and properties. In addition, compared with optical, magnetic or electric field-based rotational manipulation methods, ARM is both extremely simple and practical. The entire operation requires only a single-layer polydimethylsiloxane (PDMS) channel and a simple, low-cost piezo transducer that can be integrated to existing microfluidic platforms. Using ARM, we have demonstrated for the first time, on-chip rotation of *C. elegans*, a valuable and frequently used model organism for elucidating the molecular mechanisms underlying human diseases^46^. We have used the ARM method to analyse the morphological features of *C. elegans'* nervous and reproductive systems. Precise rotation of multicellular organisms such as *C. elegans* can provide a powerful and versatile platform to perform efficient and rapid analysis of cell and tissue morphologies or positions in three dimensions. With the growing use of organism-on-chip tools for investigating microorganisms and cells, our method is anticipated to be an invaluable tool in biology, biophysics and medicine.

## Results

### Working principle of the ARM method

**Figure 1:**
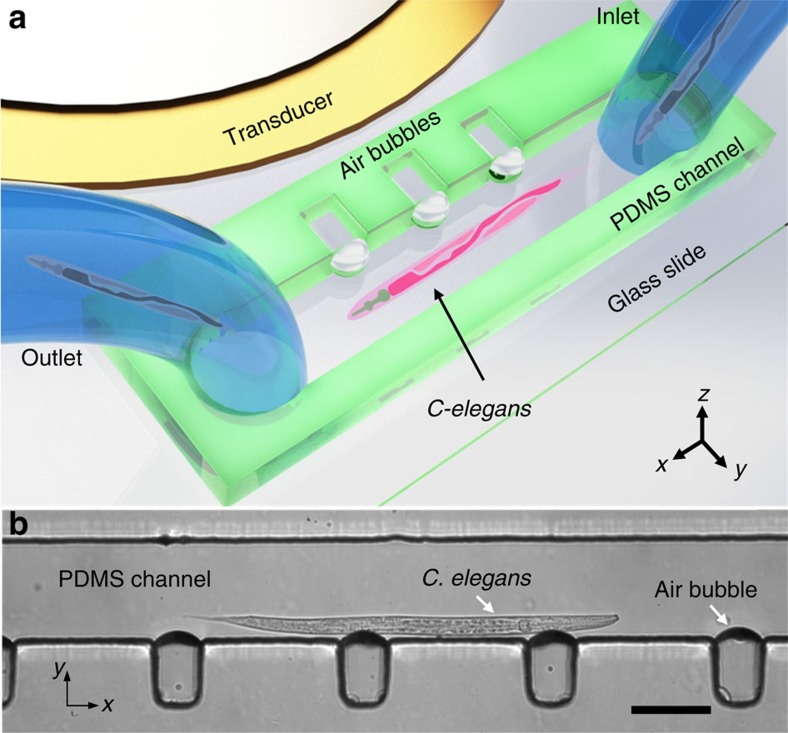
Design and operation of the acoustofluidic rotational manipulation (ARM) device. (**a**) A schematic of the experimental setup. The piezoelectric transducer that generates acoustic waves is placed adjacent to the microfluidic channel. The acoustic waves actuate air microbubbles trapped within sidewall microcavities. (**b**) An optical image showing a mid-L4 stage *C. elegans* trapped by multiple oscillating microbubbles. Scale bar, 100 μm.

**Figure 2:**
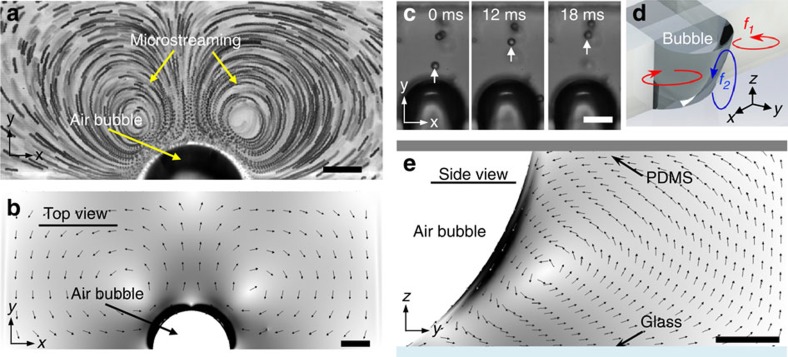
Experimental and numerical demonstration of acoustic microstreaming. (**a**) An optical image of acoustic microstreaming in the *x-y* plane during microbubble oscillation at a driving frequency of 24 kHz and voltage of 10 V_pp_. (**b**) A simulation of microstreaming from the *x-y* plane (top view) of the microbubble. (**c**) An optical image of out-of-plane (perpendicular to the *x-y* plane) microstreaming during microbubble oscillation at 25.5 kHz and 15 V_pp_. (**d**) 3D sketch demonstrating in-plane (marked in red) and out-of-plane (marked in blue) acoustic microstreaming vortices at frequency *f*_*1*_ and *f*_*2*_, respectively. (**e**) A graphic simulation illustrating microstreaming in the *y-z* plane (side view) of an asymmetric microbubble. Scale bars=30 μm. In both **b** and **e**, the arrows indicate the direction of the streaming velocity, while the colour plot shows the magnitude of the streaming velocity ranging from white (min) to black (max).

The device setup (Fig. 1a) includes a PDMS-based single layer microfluidic channel and a piezoelectric transducer. The channel contains linear arrays of rectangular microcavities (Fig. 1b) that trap air microbubbles when the liquid is injected. A piezoelectric transducer mounted on a glass slide adjacent to the channel generates acoustic waves. When the trapped microbubble is exposed to an acoustic field with a wavelength much larger than microbubble diameters, oscillations are created, which, in turn, generate acoustic microstreaming^47^ (Fig. 2a).

A spherical microbubble undergoing both radial as well as transverse oscillations in an unbounded Newtonian fluid produces a second-order steady flow that scales with the product of radial (*ɛ*) and transverse (*ɛ′*) microbubble oscillation amplitude and is linear in angular frequency, *ω*. For a fixed ratio of radial and transverse oscillations, the second-order streaming speed scales as *u*∝*ɛ*^2^*ω* (ref. 48). This scaling has been reported to be preserved even in low-symmetry cases such as a microbubble oscillating near a wall^49^,^50^, and similarly should be preserved in microcavities within our acoustofluidic channel. For such a trapped microbubble oscillating with a small amplitude *ɛ*, the flow field around the microbubble can be obtained via a perturbation expansion approach, 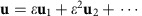. In a Newtonian fluid of density *ρ*, the oscillatory first-order velocity **u**_1_ induces a second-order steady flow (also known as the acoustic streaming) with a velocity **u**_2_ which is governed by a Stokes equation with a body force term induced by the first-order flow^51^. For an approximate semi-cylindrical microbubble oscillating in the fundamental frequency, the liquid flow pattern is characterized by two symmetric vortices in the plane of oscillation as shown experimentally and numerically in Fig. 2a,b, respectively.

A powerful feature of an oscillating microbubble in an acoustic field is that numerous modes of the microbubble can be excited to generate different microstreaming flow patterns. Our experiments demonstrated complex microstreaming patterns for higher modes of oscillation (Supplementary Fig. 1). In addition, we have observed that the acoustically excited microbubbles produce single out-of-plane microstreaming vortex (Fig. 2d and Supplementary Movie 1) as a result of microbubble shape distortion that occurs due to the difference in contact angles between the glass and the PDMS^52^, as experimentally illustrated in Fig. 2c and numerically simulated in Fig. 2e. The simulations shown in Fig. 2b,e are for the top view (that is, the *x*-*y* plane) and side view (that is, the *y*-*z* plane), respectively of acoustic microstreaming induced by the microbubble.

When particles (polystyrene, cells or organisms) are introduced near an oscillating microbubble in an acoustic field, they experience both acoustic radiation and microstreaming-induced drag forces. Radiation force on particles arises due to the scattering of the incident waves from the oscillating microbubble. The time-averaged radiation force exerted on a spherical particle due to microbubble oscillation in an acoustic field can be expressed as^53^:


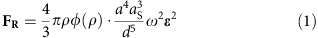



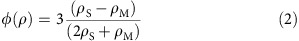


where *a*, *a*_S_, *d*, *ω*, *ɛ* are the radius of the microbubble, radius of the particle, distance between the microbubble and particle centre, angular frequency and microbubble displacement, respectively; and *ρ*_M_, *ρ*_S_ denotes the density of the surrounding liquid and density of the particle, respectively. Depending on the densities of the particle and the surrounding medium, this force can either be attractive or repulsive. Particles with density higher than that of the surrounding medium are attracted towards the microbubble (*φ*(*ρ*)>0) and particles with density lower than that of the medium are repelled (*φ*(*ρ*)>0). This expression is consistent with our experiments. For example, polystyrene particles (1.05 g cm^−3^), HeLa cells (1.04 g cm^−3^; ref. 54) and *C. elegans* (1.08 g cm^−3^; ref. 55) are attracted towards the oscillating microbubbles. In addition, the radiation force is strongly dependent on the distance between the microbubble and the particle centre, and is inversely proportional to the fifth power of *d*. However, the radiation force alone cannot explain why particles of certain diameter are not trapped.

To gain a better understanding of the trapping mechanism of particles, we have to consider the effect of acoustic microstreaming on particles. Velocity (**u**) of the acoustic microstreaming flows due to an oscillating microbubble is given by^53^:


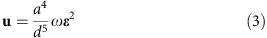


**Figure 3:**
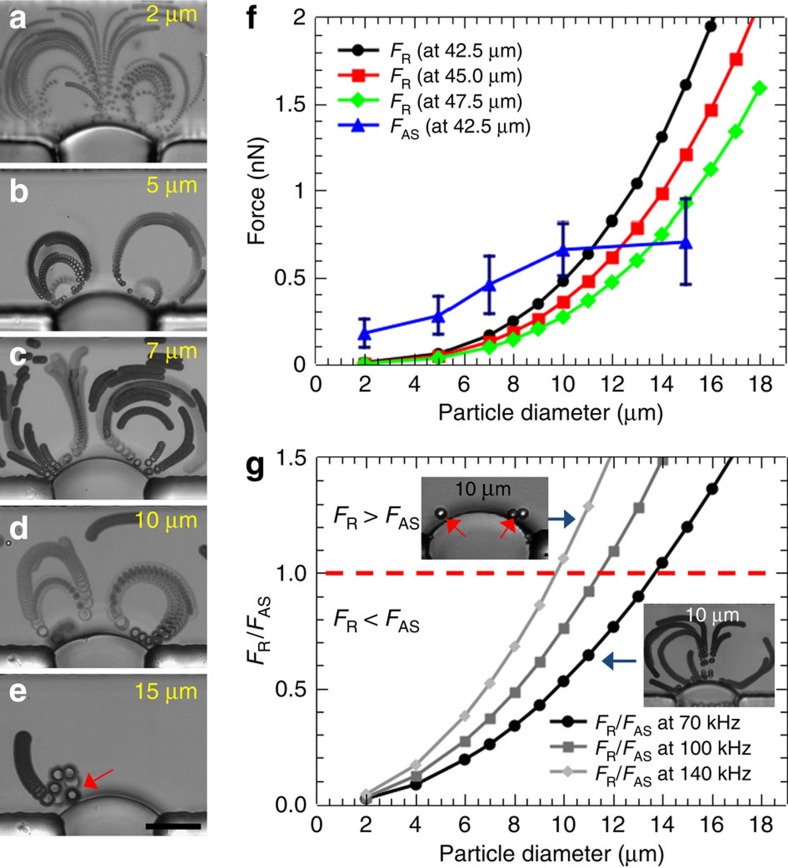
Acoustic radiation and acoustic streaming force analysis on particles with different sizes. Image sequence demonstrating (**a**–**d**) acoustic microstreaming of 2, 5, 7 and 10 μm polystyrene particles and (**e**) subsequent particle trapping of 15 μm particles at excitation frequency and amplitude of 70 kHz and ∼3 μm, respectively. (**f**) Plots of acoustic streaming (triangle, *F*_AS_) and radiation (*F*_R_) forces. The radiation forces for three different particle-microbubble centre are plotted at 42.5 (circle), 45 (square) and 47.5 μm (diamond). (**g**) The ratio of acoustic radiation to streaming forces at 70, 100 and 140 kHz excitation frequencies are plotted. The insets of **g** shows 10 μm polystyrene particles being trapped (at 140 kHz) and following the streaming flows (at 70 kHz). Error bars represent standard deviation (*n*⩾5). Scale bar, 50 μm.

The force arising from acoustic microstreaming can be estimated by the Stokes' drag:


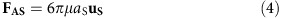


where *μ* and *u*_S_ are dynamic viscosity of the medium and the nonoscillatory velocity of the particle relative to the liquid, respectively. Figure 3a–e demonstrate the streaming effect on particles of different sizes. Although initially all the particles follow the streaming flows, 15 μm particles eventually get trapped at the microbubble surface (Supplementary Movie 2).

In general, any particles that are placed near an oscillating microbubble experience both radiation and streaming forces. To understand whether a particle will be trapped by the microbubble or not, we have to analyse the competing forces and their magnitudes. As can be seen from equation (1) and equation (4), the radiation force scales with the cube of the particle radius, while the streaming force scales linearly with the particle radius. Thus, for smaller particles, the streaming drag force dominates the motion of the particle until a transition size is reached, beyond which the radiation force becomes more dominant. Figure 3f describes the competing streaming and radiation forces for polystyrene particles of different sizes. The acoustic streaming force for particles with different sizes is calculated from the experimentally measured particle velocities. Here the distance between the microbubble and the particle centre is ∼42.5 μm. The radiation force is estimated for a microbubble acoustically excited at 70 kHz at experimentally measured *ɛ*=3 μm. The critical particle size for which the competing forces are equal can be determined by the intersection of the two plots in Fig. 3f, which corresponds to approximately 11 μm. For particles with diameter, 2*a*_S_<11 μm, F_AS_>F_R_, microstreaming dominates over the radiation forces, which results in the microparticles following the streamlines. On the other hand, for particles with diameter, 2*a*_S_>11 μm, radiation force dominates over the microstreaming force and as *φ*>0, microparticles are attracted towards the microbubble.

In addition, when the centre-to-centre distance between the microbubble and particle is increased, the radiation force weakens 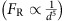, thus the threshold of critical particle size increases as marked by the intersection points of the red and green plots, as demonstrated in Fig. 3f. It is also worth noting that as the particle size increases, the streaming force is compensated by the radiation force as demonstrated by the 15 μm polystyrene particle, in *F*_AS_ plot in Fig. 3f.

A similar approach regarding particle trapping by the competing forces can also be estimated by taking the ratio of the two forces: radiation (*F*_R_) to the acoustic streaming forces (*F*_AS_)^53^:


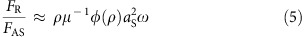


The critical particle size for which the competing forces are equal can be determined when 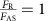, corresponding to a particle of diameter ∼14 μm. For particles lower than the transition diameter, 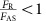, acoustic microstreaming dominates over radiation force. On the other hand, for particles greater than the transition diameter, 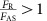, radiation force dominates and microparticles are attracted towards the microbubble as indicated in Fig. 3g. By increasing the excitation frequencies of the microbubble, it is also possible to decrease the critical particle diameter to trap smaller sized particles, as demonstrated in the inset of Fig. 3g in which 10 μm polystyrene particles are trapped at 140 kHz, whereas similar size particles follow the streamlines at 70 kHz.

We also note that when 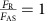, a particle is at equilibrium at some distance away from the microbubble, that is, the radiation force on a particle is balanced against the streaming force. The number of events of micro-objects remaining in equilibrium is rare; however, HeLa cells are sometimes seen to be at equilibrium, which may be attributed to its size variation. We note that the rotation of the particles is not sensitive to whether particles are trapped at the microbubble surface or trapped at equilibrium at some small distance away from the microbubble surface. The rotation is observed in either case (Supplementary Movie 3). Estimating the exact critical size is not precise based on the current particle analysis, however, it provides a design strategy to trap and rotate smaller particles. On the basis of the analysis, one would expect the transition or critical particle diameter to be similar in both analyses; however, variations may arise from the fact that the streaming-induced force is experimental in Fig. 3f and theoretical in Fig. 3g. It is also important to note that the streaming speed in equation (3) is derived for a spherical microbubble, whereas the microbubble trapped in our experiment is asymmetrical. Nevertheless, the above analyses are useful and reliable for qualitatively describing the experimentally observed particle-trapping behaviour. Finally, the above analysis is consistent with our experiment that the force generated by an oscillating microbubble is size dependent. Thus a *C. elegans* due to its much larger size experiences a larger radiation force, 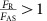, thus exhibiting a stronger trapping force. Our experiments show that a single microbubble can pull the entire mid-body of a *C. elegans* against the channel sidewall (Supplementary Fig. 2 and Supplementary Movie 4). The presence of this trapping force enables us to rotate cells and worms under 3 μl min^−1^ within the microchannels of dimensions 120 μm in width and 100 μm in depth. However, once the rotation is halted, the samples drift, which may impede proper imaging. Therefore, all the rotational experiments were performed at zero flow rate, while maintaining the pressure at the inlets and the outlets at near equilibrium.

### Rotation of microparticles and HeLa cells

Diluted microparticles were introduced near an oscillating microbubble in the microfluidic channel. The particles were attracted towards the microbubble due to the radiation force of an oscillating microbubble. Particles trapped at the microbubble surface would reposition themselves by sliding along the air–liquid interface. Observation using fast camera showed that particles are actually trapped at the nodes, the points with minimum oscillation displacement, of an oscillating microbubble (Supplementary Fig. 3). To demonstrate the node positions and particle trapping, we drove the microbubble at higher harmonics (60–90 kHz) and large driving voltage (20 V_PP_), to ensure discernable nodes and antinodes at the microbubble surface (Supplementary Fig. 3).

In a liquid, the hydrodynamic flow field produced by microstreaming induces a torque on the microparticle/cell and caused rotation. This rotation can be instantaneously turned on and off due to the low Reynolds number associated with the acoustic microstreaming. The Reynolds number for microbubble microstreaming was estimated^50^ to be 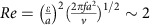, where *ɛ*=3 μm is the displacement amplitude of the microbubble oscillation, *a*=35 μm is the microbubble radius, *f*=70 kHz is the excitation frequency and *ν*=1.0 × 10^−6^ m^2^ s^−1^ is the kinematic viscosity.

**Figure 4:**
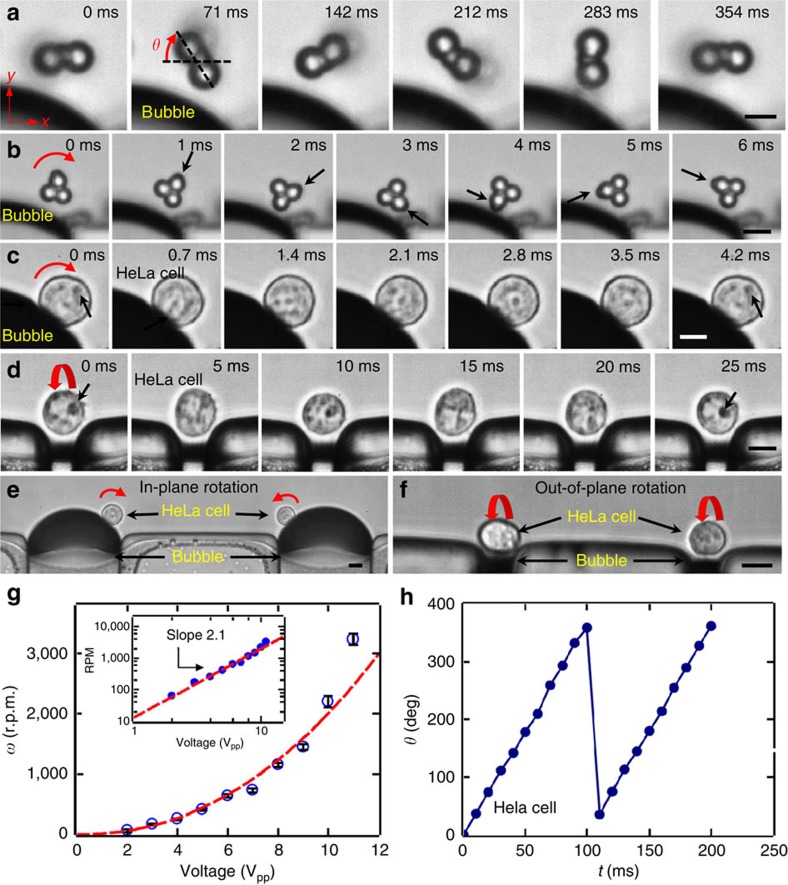
High-speed imaging showing the rotational motion of microparticles and cells caused by an oscillating microbubble. Clockwise and in-plane rotation motion of (**a**) a doublet and (**b**) a triplet. (**c**) Counter clockwise in-plane rotation of a HeLa cell. (**d**) Out-of-plane rotation of a HeLa cell. Parallel (**e**) in-plane and (**f**) out-of-plane rotation of a HeLa cells. (**g**) Plot of rotational speed *ω* against driving voltage **V**_**PP**_ of a HeLa cell driven by an oscillating microbubble, with a constant excitation frequency. The rotational rate of the cell varies as the second power of the driving voltage, *ω*∝*V*^2.1^. (**h**) Plot of the rotational angle *θ* versus a function of time *t* for a HeLa cell. Error bars represent standard deviation (*n*⩾5). Scale bars, 10 μm.

Rotational manipulation of doublets, triplets and HeLa cells were demonstrated as image sequences in Fig. 4a–c, respectively (see also Supplementary Movies 5 and 6). The torque created by an oscillating microbubble is determined by the intensity of the ambient acoustic field, which is controlled by adjusting the voltage applied to the piezoelectric transducer. Rotational rates can be as large as ∼3,000 rotations per minute in cell medium. The rotation axis of cells and particles follows the streamlines of the in-plane and out-of-plane vortices of the oscillating microbubbles and undergoes *z* axis (Fig. 4c,e) and *x* axis (Fig. 4d,f) rotation, respectively. In addition, the rotation axis is independent of the shape of the rotated object as demonstrated by *z* axis rotation of HeLa cell, doublet and triplet in Fig. 4a–c, respectively, thus making the system versatile.

The *z* axis rotation rate of HeLa cells was quantified using direct high-speed measurements of the liquid/microbubble interface. We observed that the amplitude of the microbubble oscillation is linearly proportional to the amplitude ***V*** of the voltage applied to the signal generator in water^45^,^56^. Thus, for a given microbubble configuration at a fixed excitation frequency, the acoustic microstreaming, and therefore, particle rotation *ω* attained by microbubble oscillations, should scale as *V*^2^ in water. Figure 4g shows that this quadratic relation is reasonably well satisfied by the oscillating microbubble confined in the microfluidic channel. Furthermore, it is worth noting that microbubbles excited at higher harmonics generate smooth rotational motion for cells or microparticles as demonstrated by the cycles of rotation in Fig. 4h.

A unique advantage of the ARM method is the ability to tune the rotation axis of the cells by tuning the excitation frequency (Supplementary Fig. 4 and Supplementary Note 1) or by designing bubbles with different sizes. By changing the frequency, different modes of microbubble oscillations can be achieved which results in a tuneable rotation axis as demonstrated in Supplementary Fig. 4 and Supplementary Movie 7. By changing the bubble size, out-of-plane rotation of a HeLa cell is achieved using ∼10 μm width microbubbles (keeping the channel height constant at 100 μm) as shown in Fig. 4d. This geometry yields consistent and reproducible out-of-plane rotation of cells (Supplementary Movie 8). High aspect ratio trapped bubble, oscillating at fundamental frequency, constrains its oscillations along the channel height, thus contributing to out-of-plane streaming. The rotation of the HeLa cells about the *x* axis could become useful for a thorough scan of the cell, while maintaining the focus of the microscope constant at a certain plane within the cell diameter. It is also worth noting that the trapping and rotation of particles and cells are coupled. That is, particles that are attracted by the oscillating microbubble are simultaneously being trapped by the radiation force and rotating with the streamlines of acoustic microstreaming vortices, as demonstrated by *z* and *x* axis rotations of the particles and cells.

The trapping position repeatability and rotational stability of 15 μm particles and the HeLa cells were analysed. Trapping position repeatability is characterized by measuring the arc length from the edge of the bubble to the point where the particle/cell sits (*n*⩾10 for particles and cells); arc length is measured to be 68.7±1.8 μm and 58.8±2 μm for particles and cells, respectively (Supplementary Fig. 5 and Supplementary Note 2). The small difference in the arc lengths arises from the slight curvature difference between two sets of microbubbles in water and cell medium, and can be attributed to surface tension variation between the two fluid media^57^. Positional stability analysis was performed by tracking the centre of each object (*x* and *y* coordinates) during its course of rotation (Supplementary Fig. 6 and Supplementary Note 3). Both for the cells and particles, the centre coordinates of the cell/particle were found to be within 1 μm during continuous rotational manipulation of multiple cycles. The centre positions of cells show larger scattering compared with that of the particles because of various size and shape distribution of the cells.

### Rotation of *C. elegans*

**Figure 5:**
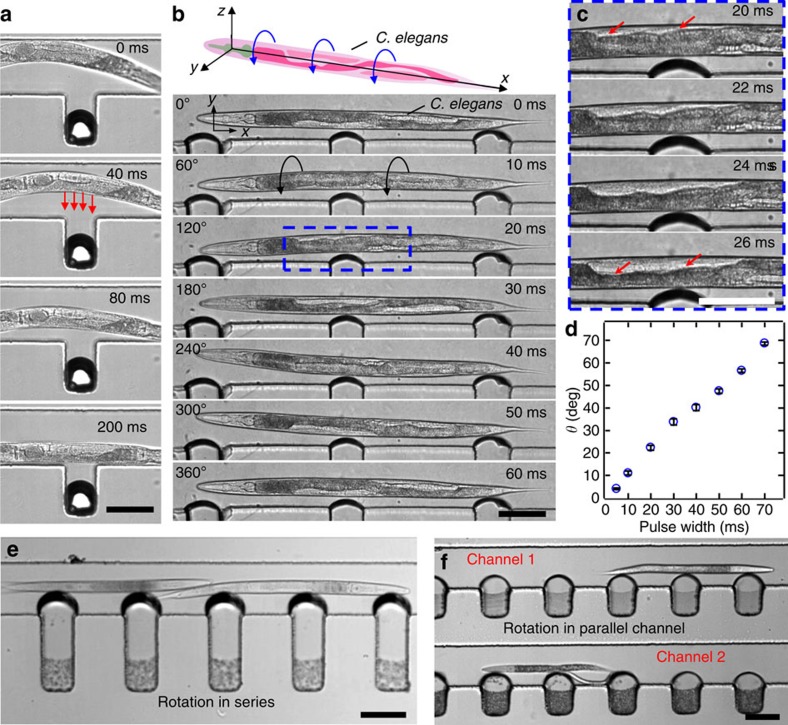
High-speed images demonstrating trapping and rotation motion of *C. elegans*. (**a**) Image sequence demonstrate trapping of *C. elegans* by an oscillating bubble via acoustic radiation force. (**b**) The rotational motion of an L4 stage *C. elegans* caused by simultaneous oscillation of multiple microbubbles. (**c**) A rotation sequence of the boxed area at a tighter time resolution. (**d**) Plot of the rotational angle *θ* versus a function of time *t* for a *C. elegans*. Error bars represent standard deviation (*n*⩾5). Scale bars, 100 μm. Trapping and rotation of multiple worms (**e**) in series arrangement in a channel and (**f**) in parallel arrangement in multiple channels.

Mixed populations of wild-type *C. elegans* at various development stages were introduced into the ARM device after they were treated with levamisole, an anaesthetic agent. During the acoustic excitation of the microbubbles at their resonances, *C. elegans* were attracted (Fig. 5a) and trapped at the surface of the microbubbles, and the excitation frequency is adjusted to out-of-plane vortex to rotate the *C. elegans* along the *x* axis. We were particular about the *x* axis rotation of *C. elegans*, which is the same as the long axis of the worm, as it allows investigating the worm at different planes. A thorough scan of the worm can be achieved for this mode of rotation axis, while maintaining the microscope focus constant within the worm diameter. Unlike cells and particles, the trapping and rotation of *C. elegans* under the current experimental setup is decoupled due to the restriction of the channel geometry and the fact that multiple bubbles are trapping the worm. Therefore, *C. elegans* cannot follow the in-plane streaming flows (*z* axis rotation), and stays trapped until the out-of-plane vortices develops by tuning the frequency (Supplementary Movie 9).

By adjusting the duration of applied power to the piezoelectric transducer, rotation of the animal could be either continuous or stepwise. Using continuously applied power at ∼92.2 kHz, the animal underwent smooth 360° rotations (Fig. 5b,c, and Supplementary Movie 10). Stepwise rotation was achieved through short pulses of acoustic excitation ranging from 5 to 70 ms durations shown in Fig. 5d for a fourth larval (L4) stage worm (see also Supplementary Movie 11). Stepwise rotation, along with voltage control, allowed repositioning of the *C. elegans* at any desired angle with excellent precision. For example, an individual worm was rotated by 4° with a 5 ms pulse as demonstrated in Fig. 5d. In Fig. 5b, a full 360° rotation occurs in 60 ms which is approximately six times faster than the stepwise rotation in Fig. 5d. For precise angular adjustment, a slow rotational rate was preferred. This could be achieved either by adjusting the applied voltage or frequency. In addition, the ARM method can be rendered for high-throughput studies as demonstrated in Fig. 5e,f and Supplementary Fig. 7. Positional stability of the worms during and after the rotational manipulation is also important for better imaging capability. We have observed slight worm drifting events along the channel once the acoustic power was turned off. This was identified as a result of the pressure fluctuations in the microchannel. By careful adjustment of the inlet and outlet tubing length and positions, and designing parallel channels (Supplementary Fig. 7) to reduce the effect of minute pressure differences in the microchannel, we achieved a more robust worm positioning once the rotation is halted (Supplementary Movie 12). In the absence of external liquid flow, the worm remains trapped and rotates in the same location after each cycle. However, during each cycle, the tip of the head of the worm rotates in an elliptical manner due to its slightly curved shape after the anaesthetic treatment. We tracked the tip of the *C. elegans* head and measured the spatial positions (*x* and *y* coordinates) during multiple-cycle rotations, also suggesting that the worm does not drift during rotation (Supplementary Fig. 6d,e and Supplementary Movie 12).

### Application of the ARM to *C. elegans* developmental studies

**Figure 6:**
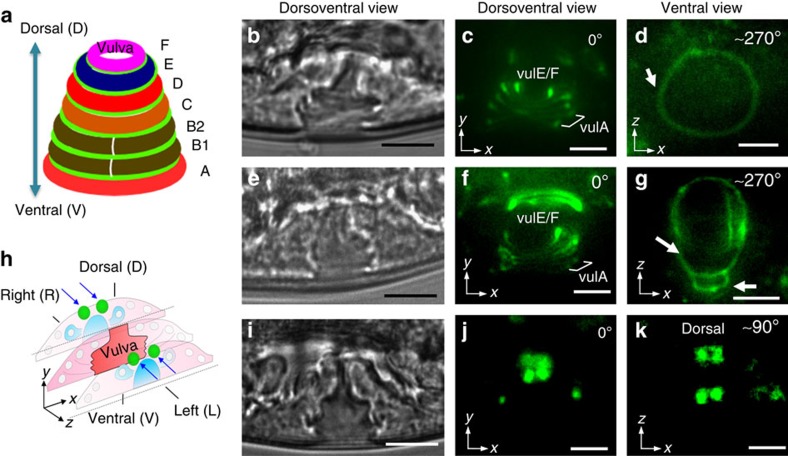
Toroid formation and cell position during *C. elegans* reproductive system morphogenesis. (**a**) A schematic of vulva structure of a *C. elegans* showing seven concentric toroidal rings, VulA to VulF along the dorsoventral positional axis, connected by adherens junctions shown in green. Digital image of a vulva of (**b**,**i**) a wild-type animal and (**e**) an *nhr-25(RNAi)* animal. Fluorescence image of toroids created by visualization of the space between *AJM-1::GFP* adherens junction rings in (**c**) a wild-type and (**f**) an *nhr-25(RNAi)* animal acoustically driven along the dorsoventral axis. (**d**) The ventral view of an ARM rotated worm showing shape and size of border of vulA in wild-type and (**g**) in *nhr-25(RNAi)* animals. In the *nhr-25(RNAi)* animal, the vulA border is expanded laterally and abnormal adherens junctions (arrows) were seen. (**h**) A schematic of vulva epithelial cell positions along the left and right axis. Two VulF cells on the right and two on the left shown in green. (**j**) Dorsoventral view shows two GFP+ cells. (**k**) Ventral view obtained through ARM clearly shows four GFP+ cells. Scale bars, 10 μm.

We used the ARM method to analyse reproductive system pathology in *C. elegans*. The nematode vulva is a passageway between the uterus and the exterior and is required for egg-laying and mating. A mature vulva is tubular in shape and is formed from 22 epithelial cells that self-organize into seven concentric rings of unique dorso-ventral positional identity, vulA to vulF (Fig. 6a). We applied ARM to examine the morphological properties of the vulva toroidal rings. We imaged transgenic animals with the adherens junction marker *ajm-1::GFP* (Fig. 6b) in which green fluorescent protein (GFP) localizes to the apical border between toroids, essentially outlining each toroid for visualization^58^. The toroids change shape and position during morphogenesis, and when comparing wild-type and mutant animals, it is important to compare similar stages. A particular stage is most easily identified from a lateral view. However, the toroid shape is most effectively visualized and compared from a dorsal or ventral view, depending on which the toroid is to be examined. We imaged a wild-type animal from the lateral side, first confirming that the animal was at the mid-L4 stage according to the characteristic vulval morphology, the extension of the gonad arms and the size of the uterine lumen (Fig. 6b). Upon fluorescence imaging along this dorsoventral axis, as seen in Fig. 6c, all the rings appear as parallel lines stacked upon one another. VulA is visible as the space between the two most ventral GFP lines, but minimal information pertinent to morphological properties of vulA, such as shape and size, is evident. Therefore, we applied ARM to rotate the animal in order to observe the complete vulA ring morphology on the ventral side. With a 270° ARM rotation, a distinct ring, defining the edge of vulA, was observed closest to the hypodermis, and its characteristic round morphology is evident (Fig. 6d).

RNA interference (RNAi) with NR4A family nuclear hormone receptors *nhr-25* results in abnormal vulval ring formation^59^. We analysed the ring morphology in *nhr-25(RNAi)* animals from both the lateral and ventral perspective for the first time. From a lateral perspective, we can identify an animal of appropriate stage, when fusions should be complete (Fig. 6e) and observe that toroids have formed (Fig. 6f). However, only after applying ARM to examine the ring morphologies from a ventral view in the same animal, can we observe an abnormally shaped toroid with defective epithelial junctions (Fig. 6g). The junction between vulA and the hypodermis is abnormally elongated in the lateral plane (arrow) and an abnormally unfused cell (arrowhead) is present. Twenty-three per cent of animals (*n*=200) have visible toroid shape defects when analysed using ARM.

As a further demonstration of the value of ARM, we also examined the expression of a GFP marker with a distinct left and right expression pattern. The epidermal growth factor homologue LIN-3 is expressed in the VulF toroid, which is comprised of two left-side and two right-side vulval epithelial cells^60^ (Fig. 6h,i). The entire *lin-3::GFP* expression pattern was effectively visualized through ARM. Along the dorsoventral axis, only two GFP+ cells were clearly seen (Fig. 6j). The ARM was applied to rotate the animal perpendicularly to the ventral position to observe all the four GFP+ cells simultaneously (Fig. 6k).

**Figure 7:**
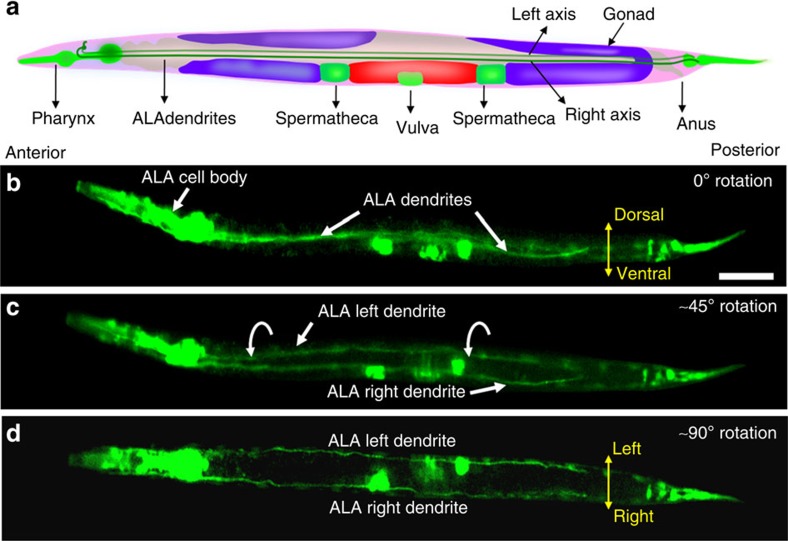
Observations of the anatomy of ALA neuron dendrites using rotational orientation. (**a**) A schematic of *C. elegans* showing the anatomy of ALA cell body and dendrites. (**b**) Fluorescence image of a *C. elegans* showing overlapped, masked ALA dendrites at the dorsoventral axis. The worm is rotated (**c**) ∼45° and (**d**) ∼90° simultaneously exposing the left and right dendrites for observation. Scale bar, 40 μm.

We also used this technique to examine the morphology of the *C. elegans* ALA interneurons using the *ida-1::GFP* transgene, which is expressed in the ALA cell body and dendrites, as well as in other neurons and the spermathecae (*ida*-1::*GFP*)^61^. The ALA cell body extends two dendrites along the left and right axis to the tail of the animal^62^ as seen in Fig. 7a. At the dorsoventral axis, it was difficult to distinguish the left and right dendrites of ALA due to masking (Fig. 7b). We used the ARM technology to rotate the animal gradually in two steps of ∼45° rotation and were able to observe the distinct migratory pattern of both the left and right ALA dendrites (Fig. 7c,d).

## Discussion

ARM provides an excellent platform for a wide range of applications in the biological and physical sciences. The ARM method can trap and rotate microparticles, cells and organisms in a compact microfluidic device by using oscillating microbubbles in an acoustic field. It is critical that the ARM method is capable of rotating micro-objects regardless of their electrical, magnetic or optical properties. Our ARM technology shows significant advances in biocompatibility and versatility beyond existing rotational manipulation methods. We have demonstrated the biocompatibility of our method by conducting a HeLa cell viability test, which resulted in an ∼99.2% survival rate for the cells after experiencing acoustic field for 1–2 min (Supplementary Fig. 8). Viability of cells in acoustic fields primarily depends on the acoustic pressure amplitude or applied voltage, and the heat associated with it. Most acoustic applications pertaining to cells in microfluidics was reported to be biocompatible and safe^21^,^63^. Unlike inertial bubble cavitation (where bubbles collapse in the presence of strong acoustic pressure) or high-amplitude strong oscillations, we used stable, low-amplitude bubble oscillations, which require very low acoustic power. The rotation of cells can even be seen at a voltage as low as 2 V_PP_ corresponding to approximately ∼60 r.p.m. Thus, low voltage corresponding to low-amplitude oscillations used for rotational manipulation does not damage cells when compared with stronger acoustic bubble cavitations^50^,^64^. The controlled rotation of HeLa cells was demonstrated by modulating the oscillation amplitude of the microbubbles. The wide range of angular velocities achieved in rotational manipulation could provide an excellent tool for massively parallel single-cell mechano-biological studies through arrays of microbubbles within horse-shoe-structures^39^,^44^,^45^ or sidewall cavities. It is an important ability to create physiological conditions to understand how cells react to mechanical forces which is critical in various applications including tissue engineering of vascular cells and heart valves^65^,^66^.

The ARM technology provides unique advantages for imaging a model organism such as *C. elegans*. During investigations of the ALA neuron dendrites, imaging in general was difficult due to overlapping of GFP patterns; rotation of the worm permitted acquisition of distinct dendrites images in a single animal and allowed access to the neuronal network of the organism. This feature holds great promise for *in vivo* laser microsurgery studies applied in axon-regeneration processes, where you can damage multiple neurons at a time and later analyse their regeneration properties with ease^10^,^14^,^17^,^18^.

Similarly, we used our method to examine the composition and structure of the *C. elegans* vulva. Rotational manipulation allowed us to categorize mutant worms by analysing the defective cell shape and size comprising the vulval rings. Specimen rotation using ARM is precise, rapid and more importantly, controllable, thus photobleaching becomes less challenging for fluorescent samples. Furthermore, dynamic rotational positioning and rapid identification of the defective cellular structures can be potentially coupled with on-chip model animal sorting applications. It is also important to note that the ARM chip costs less than $1 in bulk fabrication. The permanent instrument, which includes compact, custom-designed electronics, can be manufactured for under $100. With this inexpensive setup, even low-cost fluorescent microscopes^67^,^68^ can be used to obtain 3D imaging capability, which makes 3D imaging accessible to many low-budget laboratories around the world that do not have access to confocal microscopy facilities. The ARM method can be extended to other small organisms by simple design modifications of the microfluidic devices. The ARM method offers rapid and accurate angular adjustment of the cells and organisms. Given the growing use of organism-on-chip tools for investigating small animals, our method is valuable in the field of bioengineering, biophysics, medicine and developmental biology.

## Methods

### Device design and fabrication

A single-layer PDMS microchannel (height: 100 μm, width: 120 μm, length: 10 mm) with pre-designated microbubble trapping sites was fabricated using the soft lithography and the replica moulding technique. A silicon master mould for the microchannel was patterned using a positive photoresist (Shipley 1827, MicroChem, USA) and etched with deep reactive ion etching. The mould was then vapour coated with 1H,1H,2H,2H-perfluorooctyl-trichlorosilane (Sigma Aldrich, USA) to reduce its surface energy and any subsequent damage to the PDMS channel during the demoulding process. Sylgard 184 Silicone Elastomer Base and Sylgard 184 Silicone Elastomer Curing Agent (Dow Corning, USA) were mixed at a 10:1 (weight:weight) ratio and cast onto the silicon mould. The uncured PDMS on the silicon mould was then degassed in a vacuum desiccator for 2 h to remove any air microbubbles and later cured at 65 °C for 2 h. After gently removing the cured PDMS from the silicon mould, the inlets and the outlets were punched into the PDMS using a reusable biopsy punch (Harris Uni-Core, Ted Pella, USA). The microfluidic channel device and a 25 × 60 mm micro-cover glass (SuperSlips, VWR, USA) were treated with oxygen plasma for 10 and 60 s, respectively. Then, the PDMS device was bonded to the cover glass, and kept at 65 °C overnight. A piezoelectric transducer (model number 273-073, Radioshack, USA) was then attached to the glass slide adjacent to the channel using a thin layer of epoxy (84101, Permatex, USA).

### Device operation

The glass slide, with the attached microfluidic channel and piezoelectric transducer, was mounted on an inverted optical microscope stage (TE-2000U, Nikon, Japan). Microparticles, cells and *C. elegans* were infused into the channel through a 1-ml syringe (309659, Becton Dickinson, USA) by automated syringe pumps (Nemesys, Cetoni, Germany). Once the microbubbles were trapped via surface tension effect, the transducer was connected to a function generator to control the microbubble activation using a sine wave produced by a function generator (AFG 3011, Tektronix, USA). The driving voltages used in the experiments were 2–20 V_PP._ The working frequency for the rotational manipulation was adjusted by sweeping the frequency.

### Numerical simulation

The numerical simulations shown in Fig. 2 were performed using the open source finite element library, deal.II. Owing to the difficulties associated with obtaining a direct solution for problems involving acoustic streaming^51^, we utilized a perturbation approach to split the flow variables into the first- and second-order components. The acoustic streaming response of the fluid can be characterized by the second-order system of equations, which in turn is driven by the first-order equations. The fluid response is governed by the standard Navier–Stokes equations for a linear, viscous compressible fluid:


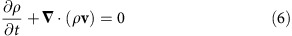



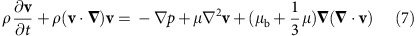


where *ρ* is the mass density of the fluid, *p* is the fluid pressure, and *μ* and *μ*_b_ are the shear and the bulk dynamic viscosities, respectively. We use Nyborg's perturbation approach^48^ where the fluid velocity, pressure and density are assumed to be of the following form:


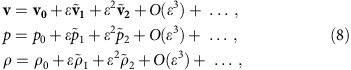


where *ɛ* is a non-dimensional parameter defined as the ratio of oscillation amplitude to the microbubble radius. Substitution of equation (8) in equations (6) and (7), and segregation of first-order terms yields a first-order system:


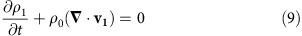



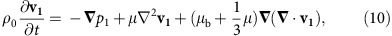


Following the same procedure for the second-order terms, and a subsequent time-averaging over a period of oscillation, yields the second-order system of equations:


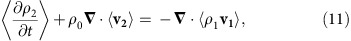



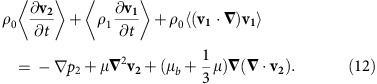


We successively solved the first- and the second-order equations to obtain the streaming velocity of the fluid. Due to the high computational costs associated with the full 3D computations of the system, we performed separate two-dimensional simulations for the top view and the side view, as shown in Fig. 2. The primary purpose of Fig. 2 is to identify the nature of the acoustic streaming field inside the microchannel. To this end, Fig. 2a,b show the top view (*x*-*y* plane) of the device experimentally and numerically, respectively, demonstrating the two vortices produced around the microbubble surface. Figure 2c shows the position of a microparticle on a focal plane close to the glass slide at different times, where the increasing distance of the microparticle from the microbubble surface demonstrates that the acoustic streaming velocity is pointed away from the microbubble surface. Figure 2d shows a sketch of the 3D nature of the flow field, which was also identified in our experiments, where we observed a single vortex in the *y*-*z* plane arising from the non-symmetric nature of the microbubble surface in this plane. The non-symmetric nature of the microbubble surface can be attributed to the fact that the top and the bottom wall of the microchannel is made of different materials (PDMS and glass, respectively), resulting in different contact angles. This results in the formation of a single vortex in the *y*-*z* plane as demonstrated by the numerical simulations for an asymmetric microbubble surface in the *y*-*z* plane (Fig. 2e).

### Image acquisition

The microfluidic cell and worm images were captured at room temperature (∼25 °C) using an inverted microscope (Eclipse TE2000-U, Nikon, Japan) with × 4, × 10, × 20 and × 60 objectives (numerical aperture: 0.45). The images were captured by Nikon imaging software (NIS-Advanced, Nikon, Japan) using a Coolsnap CCD digital camera (CoolSNAP HQ2, Photometrics, USA). Movies were recorded by a Photron FASTCAM Viewer (PFV, Photron, USA) using a fast camera (Fastcam SA4, Photron, USA). For fluorescence imaging, a Nikon filter cube (excitation: 470 nm, emission: 515 nm) and a fibre optic illumination system (Intensilight, Nikon, Japan) were used.

### Cell and particle preparation

HeLa cells (purchased from ATCC) were incubated in DMEM/F12 medium (Gibco, Life Technologies, USA), containing 10% fetal bovine serum (Gibco, Life Technologies, USA) and 1% penicillin−streptomycin (Mediatech, USA). A total 5 × 10^5^ ml^−1^ HeLa cells were suspended in MEM solution and were injected into the microfluidic channel. Polystyrene beads (polybead, Polyscience, USA) were prepared in DI water (∼10^6^ ml^−1^) at room temperature.

### Nematode strains

- N2: a wild isolate of *C. elegans* from Bristol, UK.
- BL5715: *inIs179* II; *him-8(e1489)* IV; a transgenic nematode strain carrying *ida-1*::*GFP* (*inIs179*). GFP is expressed in a subset of neurons (including ALA neurons) and the neuroendocrine uv1 cells.
- SU93: *jcIs1* [*ajm-1*::*GFP*+*unc-29*(+)+*rol-6(su1006)*] IV; a transgenic nematode strain carrying adherens junction marker −1 (*ajm-1*) fused to GFP. GFP expression is seen at the apical surfaces of cells.
- PS4308: *syIs107* [*unc-119*(+)+*lin-3(delta-pes-10)*::*GFP*; a transgenic nematode strain carrying epidermal growth factor homologue *lin-3*::*GFP*. GFP expression is seen in the anchor cell and VulF lineages during vulva development.

Strains N2, BL5715, SU93 and PS4308 were provided by the Caenorhabditis Genetics Center, which is funded by the NIH Office of Research Infrastructure Programs (P40 OD010440).

### Nematode growth and maintenance

Nematode growth medium (NGM) agar was made by dissolving and autoclaving 3 g of NaCl, 17 g of agar and 2.5 g of peptone with 975 ml of H_2_O. When the autoclaved media cooled down to 65 ° C, 1 ml of 1 M CaCl_2_, 1 ml 5 mg ml^−1^ of cholesterol in ethanol, 1 ml of 1 M MgSO_4_ and 25 ml of 1 M KPO_4_ buffer were added to it. All the strains were grown on 60 mm NGM agar petri plates at standard growth conditions of 20 °C (ref. 69). The *Escherichia coli* strain OP50 was used as the nematode food source on NGM plates.

### Levamisole treatment

An M9 solution was made by adding 3 g of KH_2_PO_4,_ 6 g of Na_2_HPO_4_ and 5 g of NaCl to 1 litre of H_2_O. The solution was autoclaved. When cool, 1 ml of sterile 1 M MgSO_4_ was added to it. A mixed staged population of *C. elegans* collected from three NGM plates was suspended in 200 μl M9. Then, 50 μl of the resulting *C. elegans* pellet was treated with 100 μl of 50 mM levamisole ([(−) tetramisole hydrochloride, Sigma-Aldrich, USA) for 15 min. The *C. elegans* were sedated with 500 μl of 100 mM levamisole just before injecting them into the microfluidic chamber.

### RNAi technique

The genes were knocked down by feeding double-strand RNA (dsRNA) to *C. elegans* in order to degrade its complimentary messenger RNA (mRNA). Briefly, to express *nhr-25* dsRNA, *E. coli* HT115 harbouring isopropyl-β-D-thiogalactosidase (IPTG) inducible T7 polymerase was grown in LB-ampicillin (50 μg ml^−1^) overnight at 37 ° C. A total 200 μl of bacterial culture was seeded onto NGM agar plates containing 50 μg ml^−1^ of carbenicillin, 12.5 μg ml^−1^ of tetracycline and 0.4 mM of IPTG. These plates were left overnight for drying at room temperature. Three or four L4-stage SU93 worms were placed on each plate and their F1 progeny were observed for vulva ring morphologies.

## Additional information

**How to cite this article:** Ahmed, D. *et al*. Rotational manipulation of single cells and organisms using acoustic waves. *Nat. Commun.* 7:11085 doi: 10.1038/ncomms11085 (2016).

## Supplementary Material

Supplementary Information Supplementary Figures 1-8, Supplementary Notes 1-3 and Supplementary References

Supplementary Movie 1 Out-of-plane streaming of particles. 5 μm polystyrene particles are used to visualize out-of-plane streaming flows at 26 kHz and 20 VPP (peak-to-peak).

Supplementary Movie 2 Effect of radiation force and microstreaming on different size particles. 2, 5, 7, 10, and 15 μm polystyrene particles are exposed to a 70 μm wide microbubble excited at 70 kHz and 10 VPP (peak-to-peak). The particles away from the microbubble experience both acoustic radiation force and microstreaming force. For smaller particles, the acoustic streaming force is dominant over the radiation force, so the smaller particles follow the streaming flows. On the other hand, 15 μm particles are trapped by the microbubble even though they follow the streaming flow initially.

Supplementary Movie 3 Trapping of HeLa cells. HeLa cells are trapped both at the nodal points on the microbubble surface and at a small distance away from the microbubble at 70 kHz and 10 VPP (peak-to-peak).

Supplementary Movie 4 Acoustic radiation force acting on a C. elegans. A smaller bubble is excited at 90 kHz and 20 VPP (peak-to-peak) in order to pull a C. elegans towards the microchannel wall close to the side-wall grove using the acoustic radiation force. Trapped bubble size can be adjusted by controlling the pressure inside the microchannel. A smaller bubble that is not protruding out from the sidewall grove is advantageous to demonstrate bubble-worm attraction without direct contact between the bubble and the worm. The movie is captured at 1,000 fps (stored by skipping every 4 frames) and is played at 30 fps.

Supplementary Movie 5 In-plane rotation of a doublet polystyrene particle. A doublet particle (~18 μm) is trapped and rotated by a microbubble excited at ~33 kHz and 10 VPP. The movie is captured at 1,000 fps and played at 30 fps.

Supplementary Movie 6 In-plane rotation of a HeLa cell. HeLa cells are trapped and rotated by a microbubble excited at 106 kHz and 4 VPP. The movie is captured at 1,000 fps and played at 30 fps.

Supplementary Movie 7 Tuning rotation axis of an egg of C. elegans. By sweeping the excitation frequency of a 70 μm wide microbubble from 60 to 90 kHz, the rotation axis of an egg of C. elegans is observed.

Supplementary Movie 8 Out-of-plane rotation of HeLa cells. HeLa cells are rotated about xaxis using high aspect ratio microbubbles with 10 μm width and 100 μm height. At high aspect ratio, microbubbles generate out-of-plane microstreaming vortices because the oscillations are restricted along the height of the microchannel.

Supplementary Movie 9 Decoupling of trapping and rotation of C. elegans. By sweeping the excitation frequency of the microbubbles from 70 to 90 kHz, a C. elegans starts rotating while initially only being trapped. In-plane microstreaming flows are also seen through the debris in the medium. Once the out-of-plane vortices are generated by tuning the frequency, the worm rotates about the x axis.

Supplementary Movie 10 Continuous rotation of an L4 stage C. elegans. An L4 stage worm is being rotated by three bubbles excited at ~92.2 kHz and 20 VPP (peak-to-peak). An entire 360° rotation takes about 60 milliseconds. A continuous sine function is used to drive the piezo transducer which yields a continuous rotation.

Supplementary Movie 11 Stepwise rotation of an L4 stage C. elegans. Multiple bubbles excited by varying duration of pulses at ~73.9 kHz and 20 VPP (peak-to-peak) are used to rotate an L4 stage C. elegans in a controlled manner. Relatively lower excitation frequencies provide a slower rotational speed for more accurate positional adjustments. Pulse duration varies from 5 milliseconds to 70 milliseconds which directly correlates to changes from smaller to larger angular steps.

Supplementary Movie 12 Parallel rotation and positional stability of C. elegans. By designing parallel microchannel and adjusting the inlet and outlet tubing, we achieved better positional stability during and after C. elegans rotation.
