# Supplementary material for: Rotational manipulation of single cells and organisms using acoustic waves
Source: Nat Commun. 2016 Mar 23;7:11085. doi: 10.1038/ncomms11085 (PMC4814581; doi:10.1038/ncomms11085)
Supplement: Supplementary Information — Supplementary Figures 1-8, Supplementary Notes 1-3 and Supplementary References [file ncomms11085-s1.pdf]

Supplementary Figures

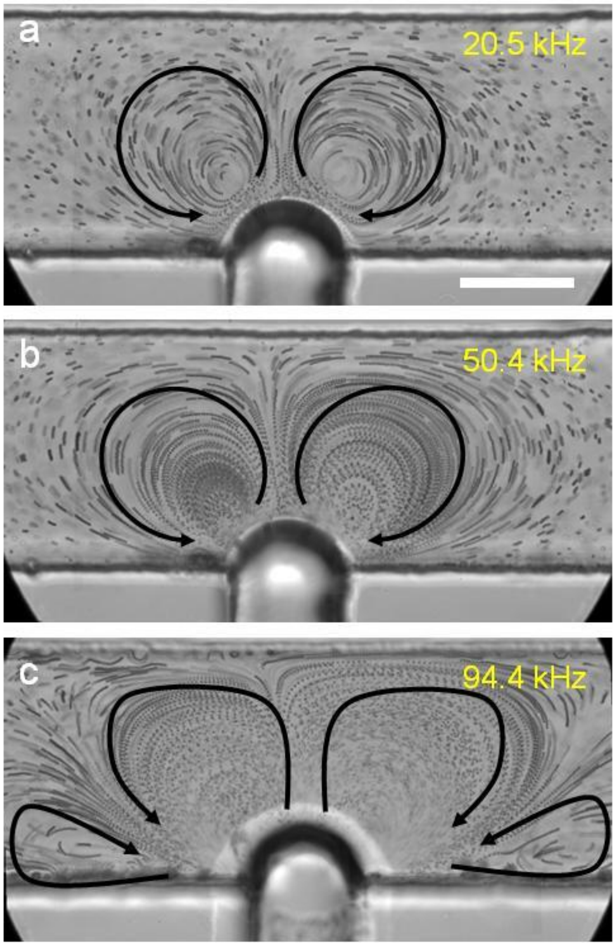

Supplementary Figure 1: Microstreaming patterns at different modes. Streaming flow patterns near a 60 μm bubble at different excitation frequencies with polystyrene tracer particles. Superimposed high-speed images of acoustic microstreaming are shown at (a) 20.5 kHz, (b) 50.4 kHz, and (c) 94.4 kHz frequencies with 5 V_{PP}. The frequency dependence of microstreaming observed here is in agreement with the previous studies[1]. Scale bar = 60 μm.

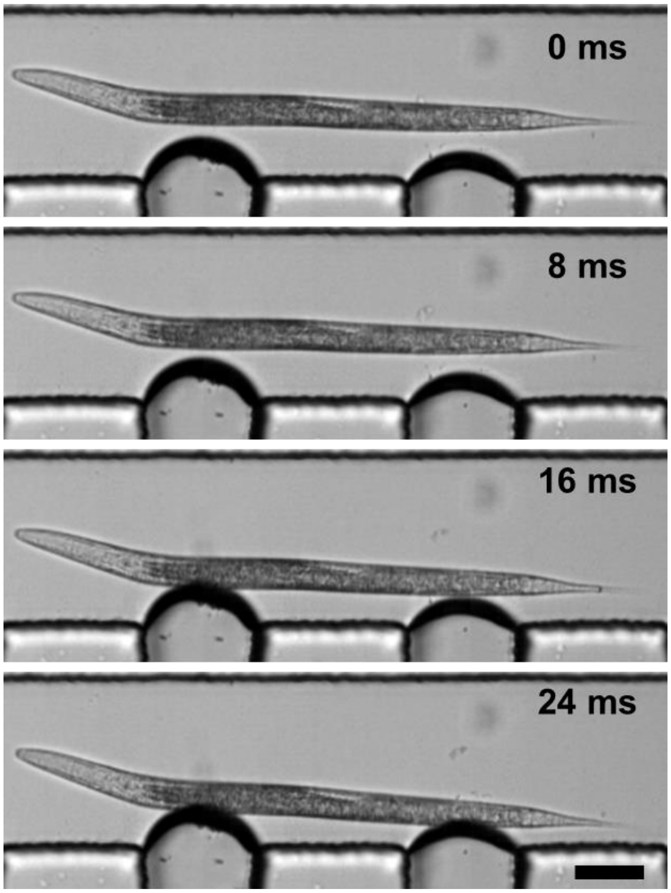

Supplementary Figure 2: Acoustic radiation force on a *C. elegans*. Image sequences shown demonstrate trapping of a *C. elegans* by oscillating bubbles where the whole body of a worm is attracted to two microbubbles within 24 milliseconds. Scale bar = 60 μm.

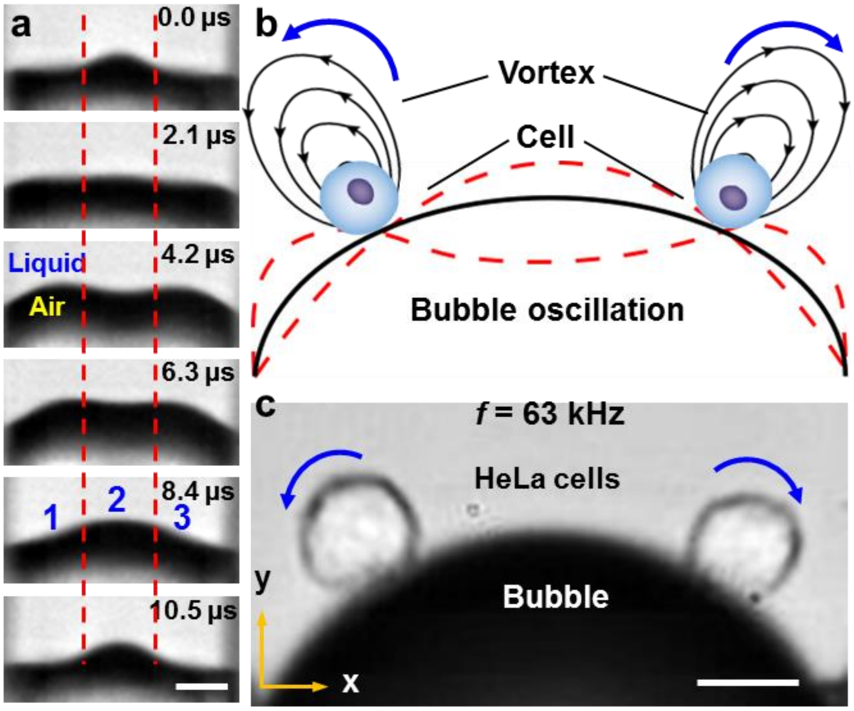

Supplementary Figure 3: Particle trapping at nodes. (a) An image sequence captured at 360,000 frames per second demonstrates bubble oscillation at 63 kHz trapped within a sidewall microcavity. The oscillation consists of multiple nodes marked by dotted red lines. (b) A cartoon schematic demonstrating cell trapping located at the nodes and the subsequent rotation during vortex generation. (c) An optical image demonstrates trapping and rotation of HeLa cells at the nodal positions of an oscillating microbubble at 63 kHz. Scale bars = 10 μm.

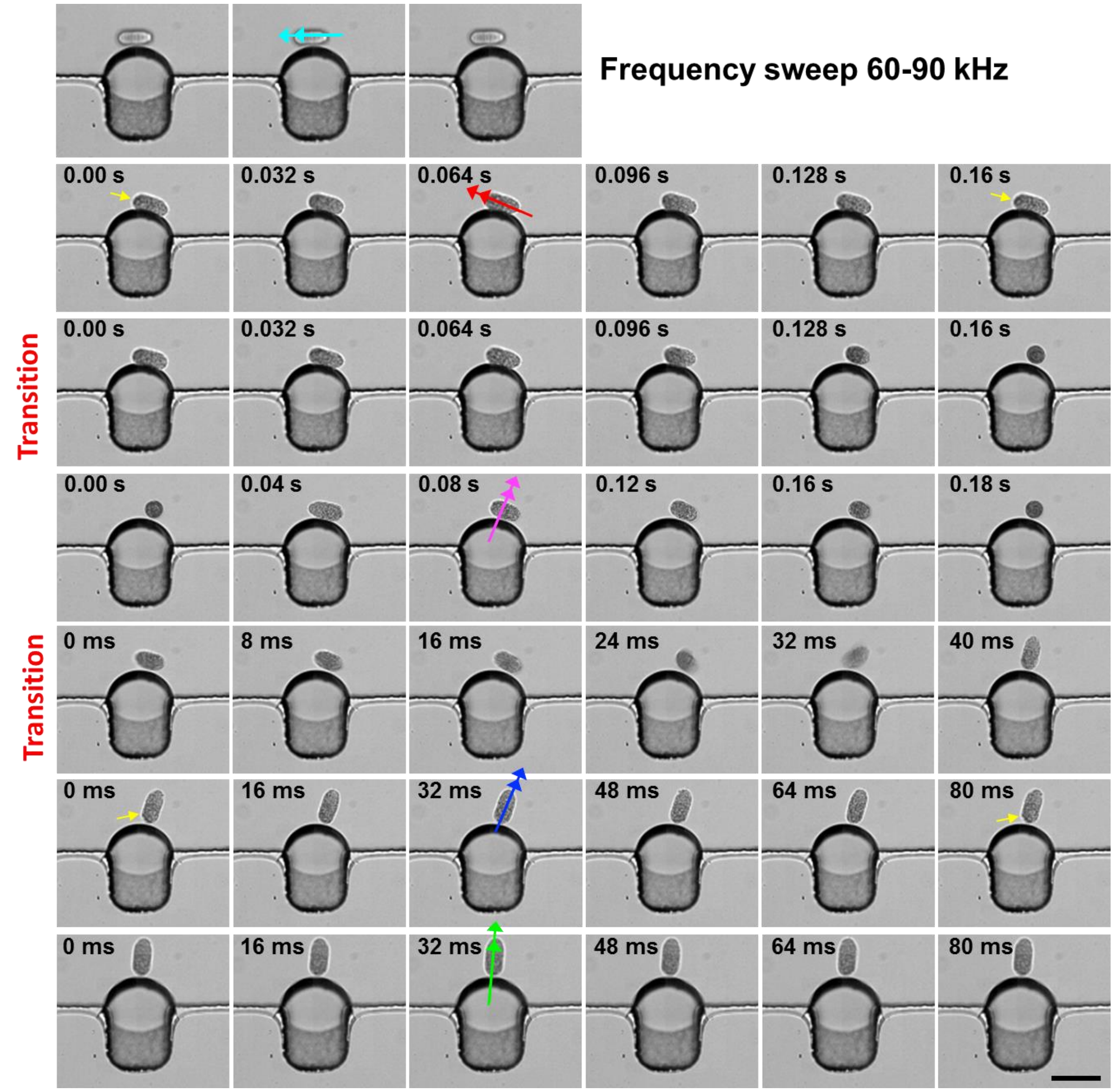

Supplementary Figure 4: Tunability of the rotation axis. Excitation frequency of a 70 μm bubble is swept from 60 to 90 kHz in order to demonstrate the change of rotation axis of a *C. elegans'* egg. During the course of its rotation, the egg switches rotation axis (marked with double headed arrows) and orientation (**Supplementary Video 7**). Scale bar = 50 μm.

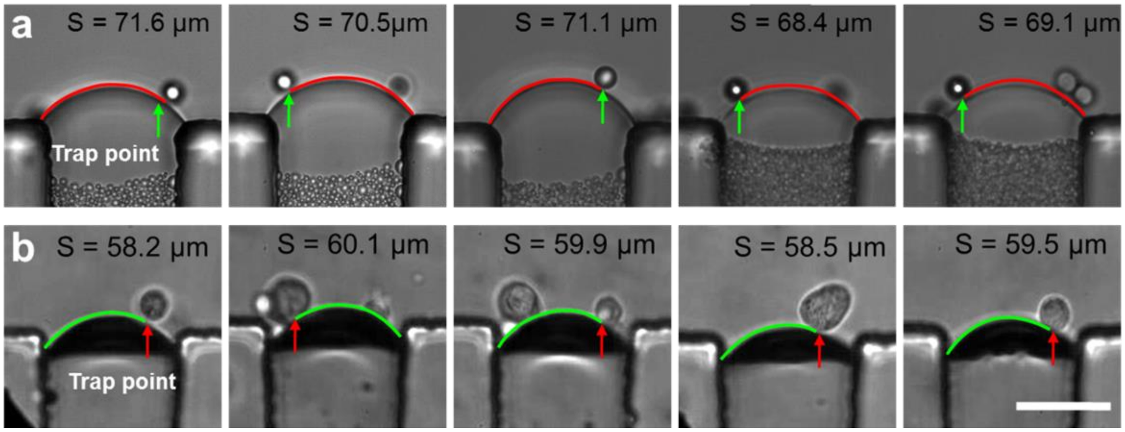

Supplementary Figure 5: Particle and cell trapping. Characterization of trapping positions with microbubbles oscillating at 70 kHz is performed by measuring the arc length drawn from the edge of the bubble to the trapping point. Trapping positions for (a) particles and (b) HeLa cells with arc length measured to be 68.7 ± 1.8 μm and 58.8 ± 2 μm, respectively ($n \geq 10$ for particles and cells). Scale bar = 50 μm.

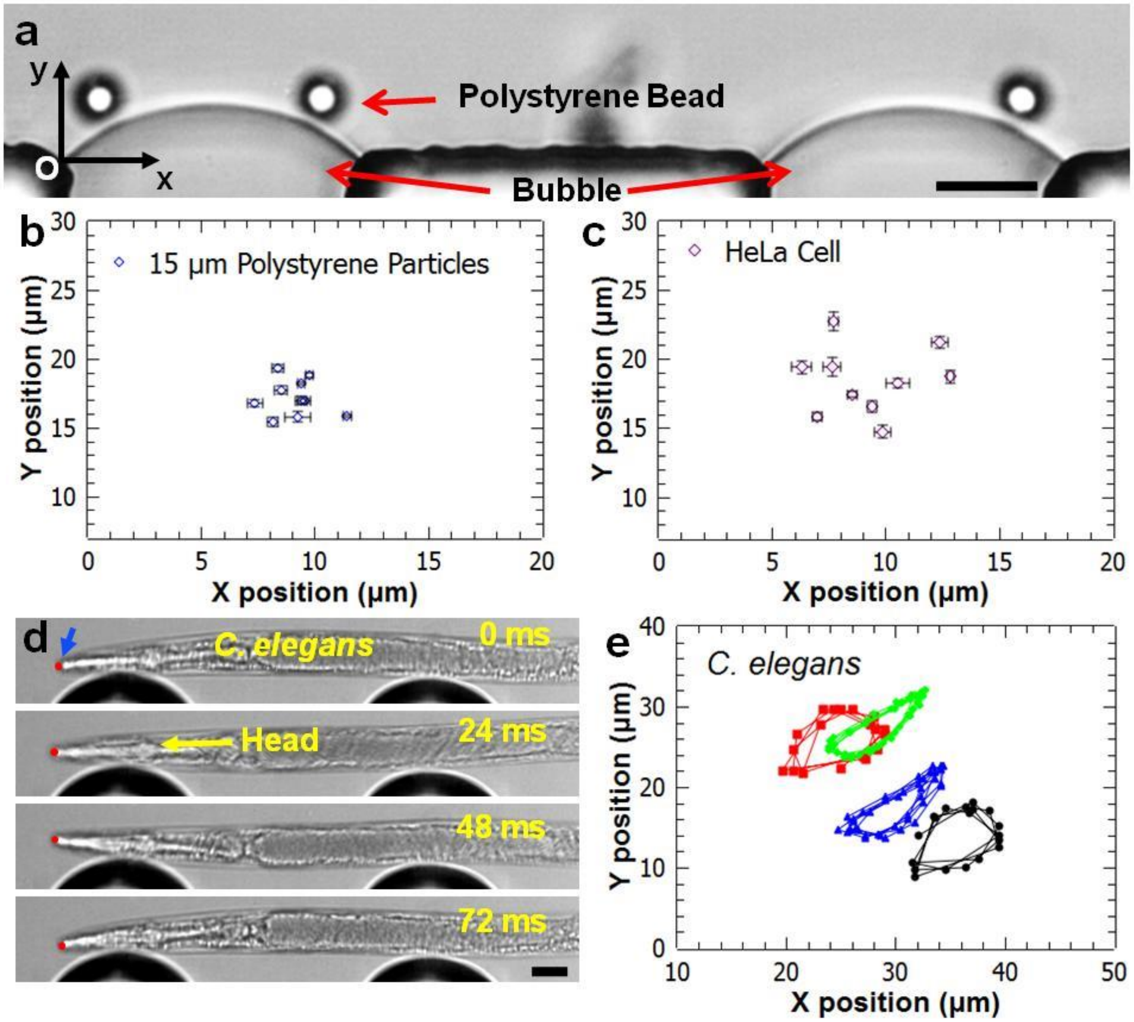

Supplementary Figure 6: Rotation stability analysis. (a) An image showing three 15 µm polystyrene particles trapped by 70 µm microbubbles. Origin point for each particle/cell is set to the edge of the microbubble for measuring the spatial coordinates during rotation. The spatial distribution of the x and y coordinates for 10 different (b) particles and (c) cells during their multiple rotations are plotted. (d) Image sequence of *C. elegans* rotation after anaesthetic treatment. Tip of the head (marked by a red dot) is tracked during multiple rotations, and the spatial coordinates are plotted in (e). Error bars represent standard deviation ($n \geq 10$). Scale bars = 25 µm.

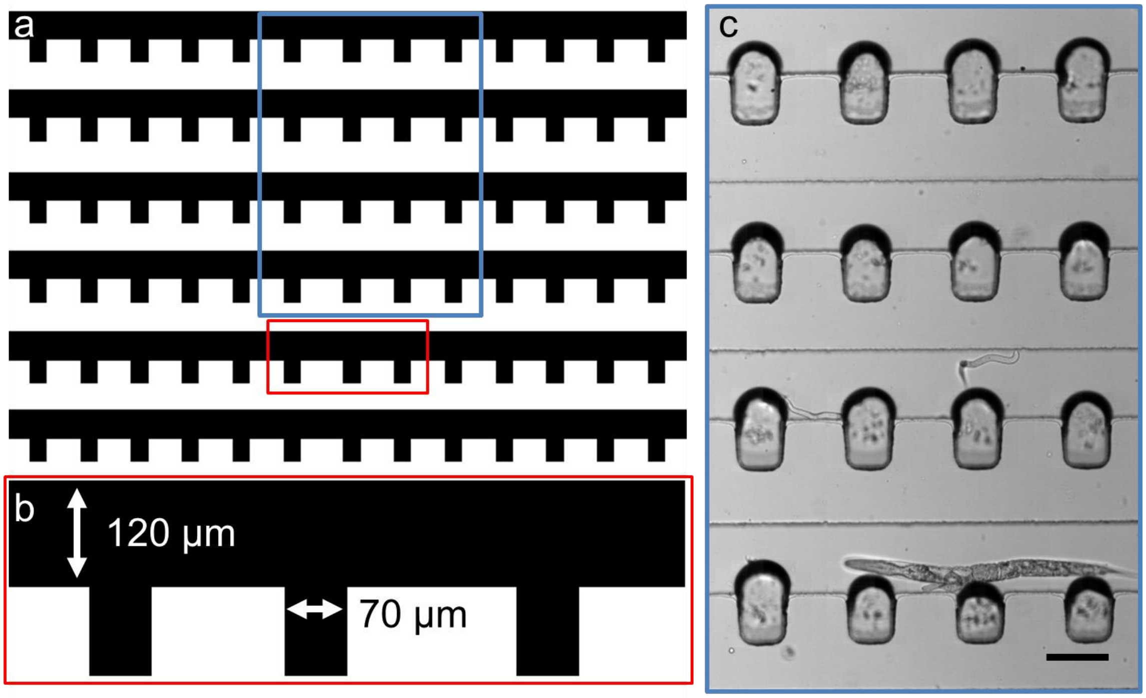

Supplementary Figure 7: Schematic and image of parallel rotation channels. (a) and (b) show the schematic of the parallel rotation device. (c) Optical image of a part of the rotation device (blue rectangle). Scale bar = 100 μm.

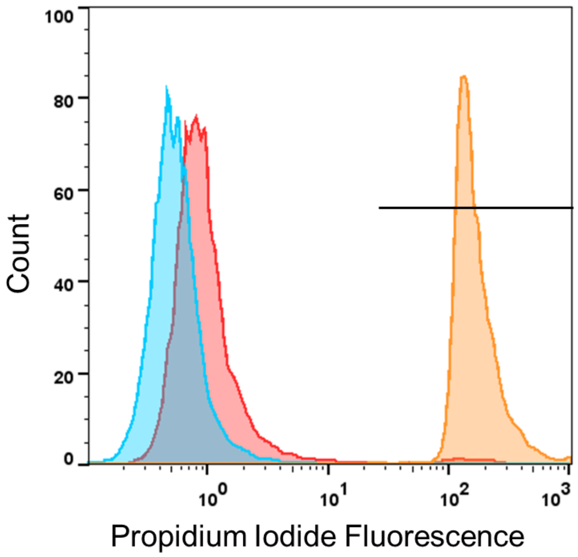

Supplementary Figure 8: Determination of HeLa cell viability. To analyse the viability of cells subjected to the ARM process, unstained HeLa cells were injected into the ARM device and acoustic microstreaming was turned on until each cell passed completely through the device (one to two minutes). These treated HeLa cells were then collected from the outlet, followed by an addition of propidium iodide (PI), which can only bind to cells if the cell membrane is damaged. The cell solution was then subjected to commercial flow cytometric analysis which indicated that 99.2% survival of the acoustically treated (red histogram) HeLa cells. The control experiments included positive control (ethanol treated and PI stained dead HeLa cells; orange histogram), and cells without microstreaming with PI addition (blue histogram). The results show that acoustic microstreaming generated with 15 V_{pp} and 60–100 kHz had no significant detrimental effect on the cells.

Supplementary Note 1: Tunability of the rotation axis

An important attribution to bubble oscillating in an acoustic field is that higher resonant modes can be excited. The modes of oscillation are primarily dependent on the applied acoustic frequency and independent of the applied acoustic pressure (or voltage). The excitation frequency (ω) and the mode (n) number are related by[2]

$$\omega_n = (n-1)(n+1)(n+2)\frac{\gamma}{\rho a^3}$$

where γ is the surface tension of the liquid. We demonstrate tunability of the orientation axis of a *C. elegans*' egg by sweeping the excitation acoustic frequency from 60 to 90 kHz (**Supplementary Video 7**). The video demonstrates that the change in rotation axis is not continuous but rather discretized. This is expected since the rotation occurs predominantly at the harmonics and the harmonics of the oscillating bubble occurs at certain frequencies.

Supplementary Note 2: Particle and cell trapping repeatability

The trapping position for particles/cells is characterized by measuring the arc length along the air-liquid interface from the edge of the bubble to the trapping point, (**Supplementary Figure 5a and 5b**). We found that the trapping positions for the particles and cells to be 68.7 ± 1.8 µm and 58.8 ± 2 µm, respectively. The variation in the arc length for the particles (**Figure 5a**) and the cells (**Figure 5b**) arises due to change in bubble shapes in DI water for particle trap and DMEM/F12 (Dulbecco's Modified Eagle Medium) for cell trap, and can be can be attributed to surface tension dissimilarity between the two fluid media[61].

Supplementary Note 3: Rotational stability analysis of particles, cells, and *c. elegans.*

For rotational stability analysis, we manually tracked the spatial positions (*x* and *y* coordinates) of the particles/cells' center during rotation ($n \geq 10$ for particles and cells), as shown in **Supplementary Figure 6a-c**. For each particle, we measured the mean and standard deviation during its rotation of multiple cycles. Error bars (*i.e.*, the standard deviation) demonstrates the stability during rotation of each particle. Similarly, we measured the mean and standard deviation of the center position of the HeLa cells. For the slightly elongated HeLa cells, the center point was taken as the intersection of major and minor axes of the cells. The scattering of the center positions of the HeLa cells is higher than that of particles, and can be attributed to various size and shape distribution of the cells. Nonetheless, the x and y positions of each cell/particle remain within 1 μm during rotation.

For *C. elegans*, trapping is usually carried out by multiple oscillating bubbles. As long as the worm is in close vicinity of the oscillating bubbles, it can get trapped, which can then be rotated by tuning the excitation frequency. *C. elegans* is infused into the channel *via* an external precision pump, which has the capability to position the worm in close vicinity of the bubbles. In the absence of external liquid flow, the worm remains trapped and rotates in the same location after each cycle. However, during each cycle, the tip of the head of the worm rotates in an elliptical manner due to its slightly curved shape after the anaesthetic treatment. We tracked the tip of the *C. elegans* head and measured the spatial positions (*x* and *y* coordinates) during rotation of multiple cycles, as demonstrated in **Supplementary Figure 6d and 6e**. These results suggest that the worm does not drift during rotation.

Supplementary References

1. Wang, C., Rallabandi, B. & Hilgenfeldt, S. Frequency dependence and frequency control of microbubble streaming flows. *Phys. Fluids* **25,** (2013).

2. Leighton, T. G. *The acoustic bubble*. (Academic Press, 1994).
